# Supplementary figures and images for: MicroRNA-99 family in cancer: molecular mechanisms for clinical applications
Source: PeerJ. 2025 Mar 27;13:e19188. doi: 10.7717/peerj.19188 (PMC11955196; doi:10.7717/peerj.19188)

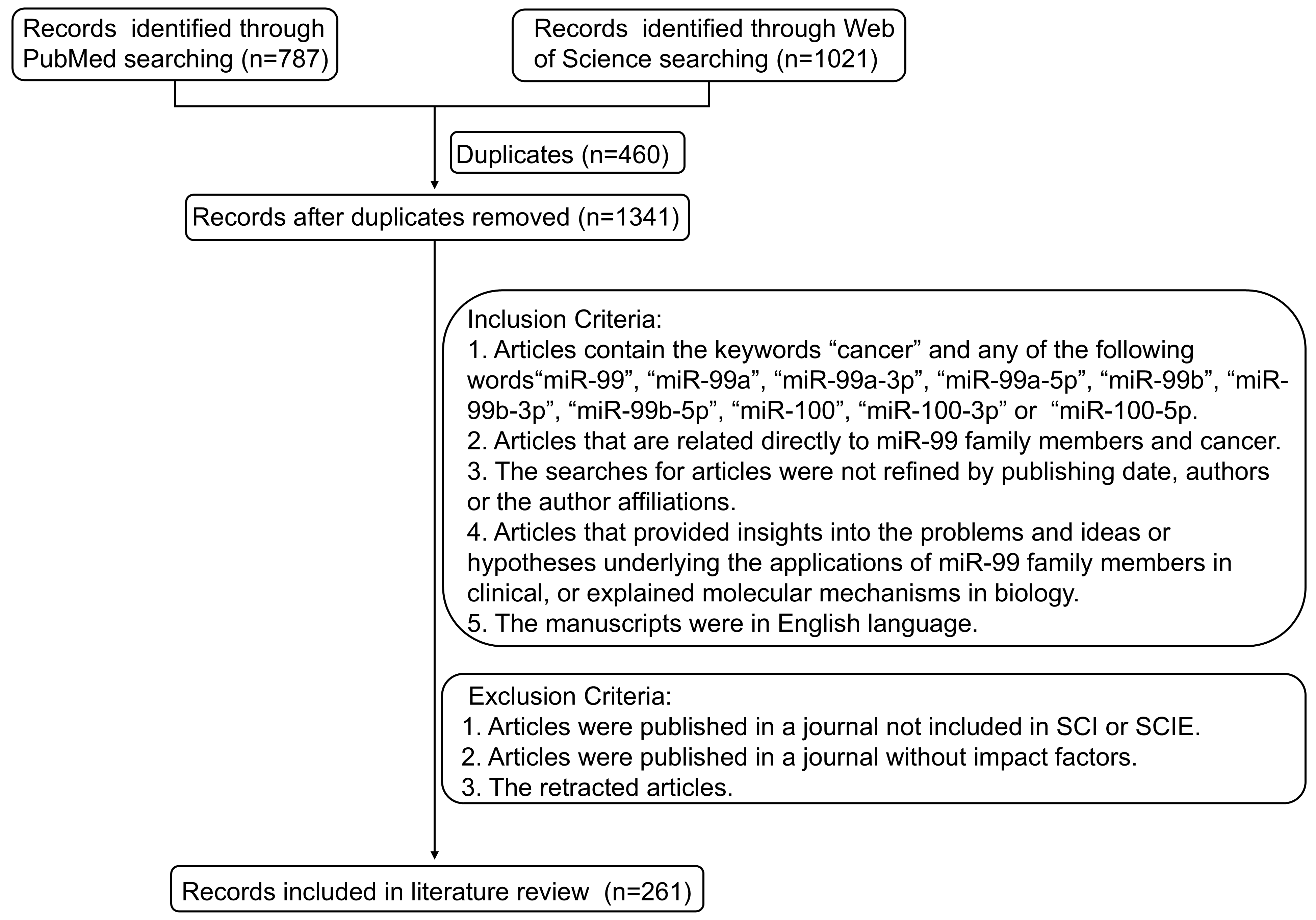

Supplement: Supplemental Information 1 [file peerj-13-19188-s001.png]
